# Effects of Supplementation With Antioxidant Agents on Sleep in Autism Spectrum Disorder: A Review

**DOI:** 10.3389/fpsyt.2021.689277

**Published:** 2021-06-28

**Authors:** Elena Zambrelli, Althea Lividini, Sofia Spadavecchia, Katherine Turner, Maria Paola Canevini

**Affiliations:** ^1^Childhood and Adolescence Neuropsychiatry Unit, Epilepsy Center–Sleep Medicine Center, Azienda Socio-Sanitaria Territoriale Santi Paolo e Carlo, San Paolo Hospital, Milan, Italy; ^2^Department of Health Sciences, University of Milan, Milan, Italy

**Keywords:** sleep, autism spectrum disorder, melatonin, L-carnosine, luteolin, antioxidant, coenzyme Q10, quercetin

## Abstract

Autism spectrum disorder (ASD) is a heterogeneous neurodevelopmental condition, whose etiology remains poorly understood in most cases. Several genetic, epigenetic and environmental factors have been implicated in ASD pathogenesis and numerous studies have provided evidences for increased levels of oxidative stress and reduced antioxidant capacity in patients with ASD. Recent clinical trials explored supplementation with antioxidant agents as a potential therapeutic strategy for ASD, investigating the impact of this treatment on behavioral symptoms and on most common comorbidities of the disease, including sleep disturbances. Among all medical conditions associated to ASD, sleep problems are highly prevalent and are supposed to be positively related to the severity of the disease. Moreover, studies on animal models support the hypothesis of a relationship between oxidative stress and sleep deprivation. The aim of this review is to summarize the current state of the literature on the effect of antioxidant treatment on sleep disturbances in patients with ASD. Twenty-one articles were included in final synthesis. Of them, 15 studies involved Melatonin, 1 Tryptophan and 5 focused on supplementation with other antioxidant agents (namely Coenzyme Q10, L-Carnosine, Luteolin and Quercetin). Despite the high prevalence of comorbid sleep troubles in ASD, there is a paucity of data on the efficacy of antioxidant agents in those patients. Further research is needed to better define the role of antioxidants agents as adjunctive therapy in the management sleep disorders in children and adolescents affected with ASD.

## Introduction

Autism Spectrum Disorder (ASD) consists of a group of lifelong, heterogeneous neurodevelopmental disabilities, characterized by early-onset, persistent impairments in social interaction and communication, and by restricted, repetitive patterns of behavior, interests and activities, not better explained by intellectual disability or global developmental delay (1). Despite the large amount of research in the field, ASD etiology still remains poorly understood. Several genetic, epigenetic and environmental (pre-peri-postnatal) factors have been associated to higher risk of developing ASD (2, 3).

Oxidative stress-related metabolites have been suggested as potential biomarkers of the disease, given the particular vulnerability of the central nervous system (CNS) to the effects of oxidative imbalance (4). High energy needs, with excessive production of reactive oxygen species (ROS) and relatively small total antioxidant capacity are among the causes of brain specific susceptibility to oxidative agents (2, 4). Many reports showed alterations in the activity of cellular anti-oxidative defense mechanisms in patients with ASD (5), while lower plasma levels of homocysteine, carnosine, methionine, cystine and threonine were found in children with ASD compared to typically developing children (6). Interestingly, various studies reported high levels of oxidative stress in cortical areas involved in speech processing, memory, social interaction, and sensory and motor coordination, functions that are typically impaired in ASD (7). Moreover, improvements in the behavior of individuals with ASD have been observed after receiving antioxidant treatment, including vitamin C, vitamin B6, N-Acetylcysteine, magnesium and others (7, 8).

Reduction in antioxidant enzyme and glutathione levels have also been associated to sleep deprivation in animal models, with normalization of antioxidant content after recovery sleep, suggesting that sleep loss promotes cellular oxidative stress (9, 10).

Among all medical conditions associated to ASD, sleep problems rank as the most common, with an estimated prevalence varying from 40 to 80% (11). These patients experience a wide range of sleep disturbance, with sleep-onset insomnia (increased sleep latency or time to fall asleep) and sleep-maintenance insomnia (decreased sleep duration, decreased sleep continuity, and increased and early awakenings) resulting among the most common sleep problems reported in ASD (12). Several investigations reported that insufficient/disturbed sleep accounts for between 22 and 32% of the variance of behavioral symptoms in children with ASD (13) and is positively related to the severity of ASD (14, 15). On the other hand, ASD core symptoms may undermine social-related circadian routine and interfere with bedtime, worsening sleep problems; these evidence suggest a bidirectional relationship between sleep and behavioral dysregulation in ASD (16).

While the pharmacological treatment of sleep disorders in ASD children has been extensively reviewed, non-pharmacological interventions and nutritional supplements have been relatively under-examined (17). A separated discussion is required for melatonin, a hormone principally secreted by the pineal gland with an endogenous, circadian rhythm influenced by light/dark conditions. Extensive researches indicate that melatonin not only plays a key role in regulating sleep/wake cycle, but it also has potent antioxidant and anti-inflammatory properties and it is involved in the immune response and neuroprotection (18, 19). Prolonged-release Melatonin received European Medicines Agency approval for treatment insomnia in children and adolescents (2–18 years old) with ASD or Smith-Magenis Syndrome.

To summarize, current evidences support the hypothesis that oxidative stress could contribute to the development of ASD and researchers have shown growing interest toward the therapeutic potential of antioxidative agents in the disease. Despite that and despite the high prevalence of sleep problems in patients with autism, studies investigating the beneficial effects of antioxidant supplementation in ASD only seldomly report data on sleep or include improvements in sleep disturbance among treatment end-point (20–22).

The aim of the present review is to summarize the current state of the literature on the effect of antioxidative treatment on sleep disturbances in patients with ASD, also highlighting the aspects that require further investigations.

## Methods

A systematic search of the literature was performed using PubMed and Embase, last accessed on January 31th 2021. The following keywords and search strategy were selected: [“antioxidant” OR “oxidative stress”] AND [“autism”] AND [“sleep”], appearing in all fields.

The results of this search were imported in Mendeley and duplicates were removed automatically, leading to a list of one hundred and fifty-five titles. Abstracts of the papers were reviewed according to the following inclusion and exclusion criteria.

Inclusion criteria were: (1) articles from peer-reviewed literature containing original data; (2) written in English.

Exclusion criteria were: (1) articles include no data on humans; (2) not written in English; (3) review papers, meta-analyses, case reports, editorials, conference abstracts, letters to the editor, (4) articles include no data on sleep (subjective or objective) measures; (5) trial does not involve antioxidant supplementation; (6) articles judged to be non-relevant to the purpose of this review.

**Figure 1:**
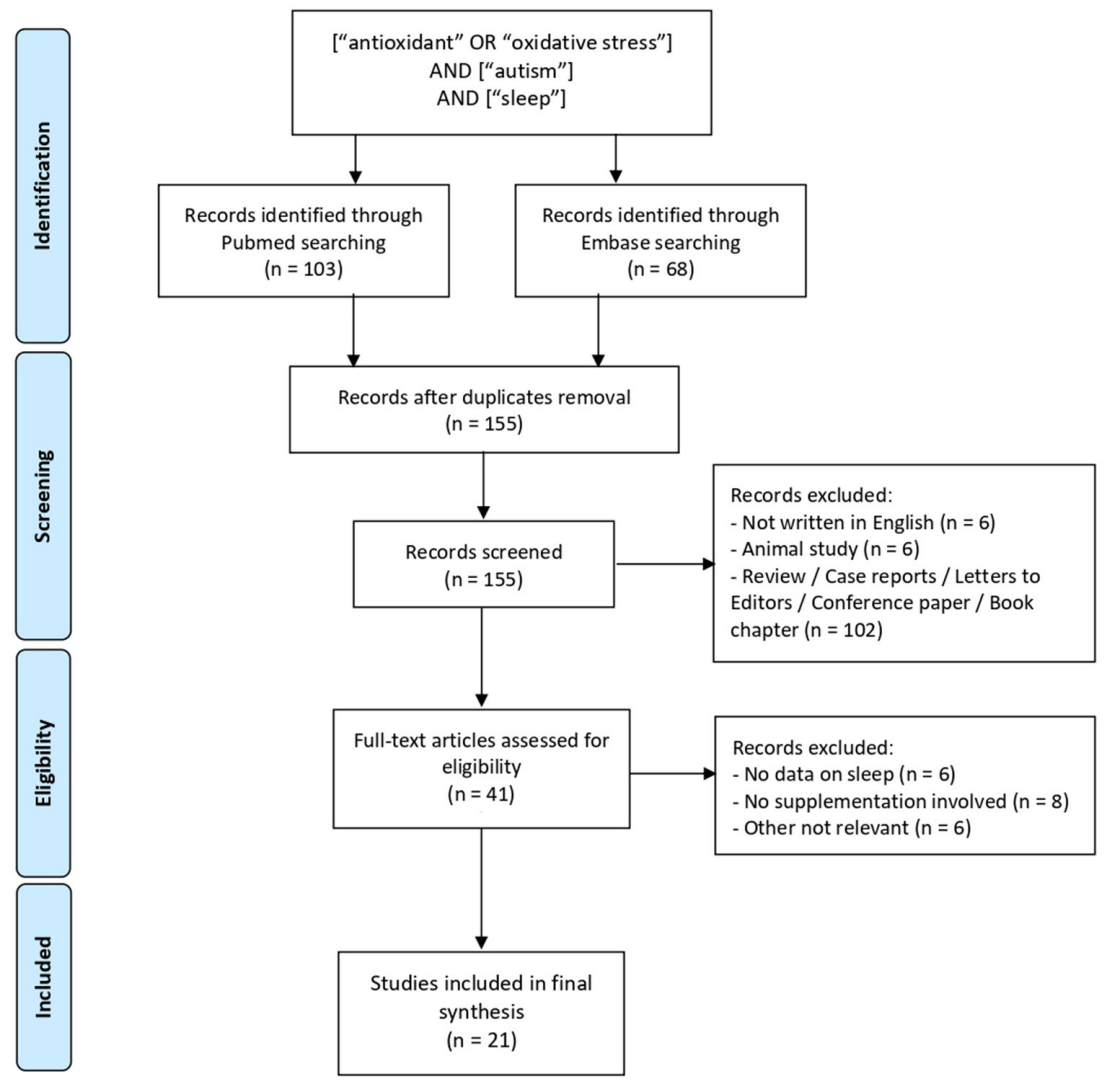
Preferred reporting items for systematic reviews and meta-analyses (PRISMA) flow diagram of literature review and study selection.

Of the one hundred and fifty-five titles initially yielded, twenty-one articles met final inclusion criteria (Table 1). Two authors (A.L., S.S) reviewed abstracts and full text for inclusion in final review. Figure 1 summarizes this iterative process, carried out in accordance with the PRISMA guidelines (43).

**Table 1:** Articles investigating the effect of antioxidant agent supplementation on sleep disturbances in children with ASD, included in the final synthesis.

**References**	**Sample size**	**Age (min-max, y)**	**Antioxidant agent (treatment duration, dose)**	**Study design**	**Main results**
**(A) ANTIOXIDANT AGENTS OTHER THAN MELATONIN**					
Ann Abraham et al. (23)	63 (2 groups: L-Carnosine = 32; Standard care (occupational and speech therapies = 31)	3–6	L-Carnosine (2 months, 10–15 mg/kg/day, twice a day)	Randomized, prospective, open-label, parallel-group trial	No improvement in sleep disturbances as assessed by BEARS sleep screening tool, fulfilled by parents, in both groups. “Intellectual response” in terms of attention statistically improved in the intervention care compared to the standard care group. No improvement in any other outcome measures (total score of CARS2-ST, ATEC and 6-GSI), as assessed by clinical psychologist and/or reported by parents, in both groups.
Mehrazad-Saber et al. (24)	43 (2 groups: L-Carnosine = 21; Placebo = 22)	4–16	L-Carnosine (2 months, 500 mg/day, once a day)	Double-blind, randomized and placebo-controlled trial	Significant reduction in “Total sleep disorders,” “Sleep duration” and “Parasomnias” scores, as assessed with CSHQ (fulfilled by parents), in L-Carnosine group, compared to placebo group. No significant difference in autism behavioral outcome in L-Carnosine group compared to the control group.
Mousavinejad et al. (25)	78 (3 groups: High dose = 26; Low dose = 26; Placebo = 26)	3–12	Coenzyme Q10 (Ubiquinone) (3 months, High dose = 60 mg/day, Low dose = 30 mg/day, once a day, preferably with lunch meal)	Randomized, parallel, placebo-controlled trial	Significantly higher rate of improvement in sleep disorders, as assessed with a Global Impressions parental questionnaire, in the patients treated with high-dose Coenzyme Q10 compared to the low-dose and the placebo groups.
Gvozdjáková et al. (26)	24	3–6	Coenzyme Q10 (Ubiquinol) (3 months, 50 mg/day, once a day for 1 week, then 100 mg/day, twice a day)	Clinical trial	Parents reports indicated an improvement in sleep disturbances in 34% of patients after treatment with Ubiquinol [Improvements were reported also in verbal communication (21%) and playing games of children (42%)].
Taliou et al. (27)	40	4–10	Luteolin and Quercetin (26 weeks, respectively, 10 and 7 mg/kg/day)	Prospective, open-label pilot trial	Significant improvement in adaptive functioning, as measured by VABS scores (parent interview), and a reduction in ABC subscale scores were found after treatment with Luteolin/Quercetin. Sleep not investigated as a treatment outcome. Sleeping difficulties were reported by parents in 3/40 patients after treatment.
**References**	**Sample size**	**Age (min-max and/or medium** **±** **SD, y)**	**Formulation, treatment duration, dose**	**Study design**	**Main results**
**(B) MELATONIN**					
Yuge et al. (28)	74 (Total sample = 93, including NDD other than ASD)	6–1510.4 ± 2.5	Immediate-release Melatonin, 26 weeks, 1, 2 or 4 mg/day (increased/decreased dose based on clinical condition) Of note: Melatonin treatment was combined with adequate sleep hygiene interventions	Multicenter, collaborative, uncontrolled, open-label, phase III clinical trial	SOL shortened significantly in the medication phase I compared to baseline in patients with ASD (and in patients with the other NDD investigated) and persisted in the medication phase II and the follow-up phase. “Refusal to going to bed at prespecified bedtime,” “temper upon wakening” and “sleepiness after awakening” (qualitative variables) improved significantly in the medication phase I from baseline and persisted in the follow-up phase in all patients (subgroup analyses not shown). SOL and qualitative variables were recorded with the electronic sleep diary, filled in by caregivers. Melatonin treatment was well-tolerated.
Schroder et al. (29)*	95 (PedPRM = 51; placebo = 44)	2–17.5	PedPRM, 91 weeks, 2, 5, or 10 mg/day	Randomized, placebo-controlled double-blind multicentric study	PedPRM treatment resulted in significant improvement in externalizing but not internalizing behavior, as assessed by SDQ, compared to placebo. PedPRM treatment was associated with an improvement in parents' WHO-5-assessed quality of life and PSQI-assessed caregiver's sleep quality compared to baseline PedPRM was generally safe; in the double-blind phase somnolence was more commonly reported with PedPRM (28.3%) than placebo (10.8%).
Maras et al. (30)*	95 (PedPRM = 51; placebo = 44)	2–17.5	PedPRM, 39 weeks, 2–5–10 mg/day	A prospective, open-label follow-up study	Significantly increased TST, reduced SOL, longer uninterrupted sleep period, improved quality of sleep and reduction in mid-sleep awakenings (all assessed with SND) were reported in patients treated with PedPRM. PedPRM was generally safe; most frequent treatment-related adverse events were fatigue (5.3%) and mood swings (3.2%).
Gringras et al. (31)*	125 (ASD = 121, SMS = 4) (PedPRM = 60; placebo = 65) Of note: sleep failed to improve on behavioral intervention alone	2–17.5	PedPRM, 13 weeks, 2–5 mg/day	Randomized double-blind, placebo-controlled study	Significantly higher TST and decreased SOL, as assessed by SND, were found in patients treated with PedPRM compared to the placebo group. The PedPRM-treated group showed a greater improvement (decrease) in total CSDI score compared to placebo-treated group. PedPRM was generally safe, with somnolence being more commonly reported with PedPRM than placebo.
Ayyash et al. (32)	9 (Total sample = 45, including 29 intellectual disability; 7 ADHD)	1.7–6	Immediate-release Melatonin, 326 days (mean duration) 2, 5–10 mg/day (doses increased according to response)	Prospective observational naturalistic study	Thirty-eight percent of children responded to low (2.5–3 mg), 31% to medium (5–6 mg) and 9% to high doses (9–10 mg) of melatonin, with a significant increase in total hours of sleep/night, decreased sleep onset delay and decreased number of awakenings/night, as measured with sleep diaries. Melatonin is generally effective and safe in children with neurodevelopmental conditions.
Goldman et al. (33)^†^	9 (subgroup of 24)	3–8	Immediate-release Melatonin, 3 weeks, 1–9 mg/day	Open-label dose-escalation study	SOL, as measured by actigraphy, improved with supplemental treatment. Night wakings, based on CSHQ, showed an improvement with treatment and their number did not increase toward morning despite short duration of action of supplemental melatonin.
Malow et al. (34)^†^	24	3–10	Immediate-release Melatonin, 14 weeks, 1–9 mg/day	Open-label dose-escalation study	Supplemental Melatonin improved SOL, as measured by actigraphy, in most children at 1 or 3 mg dosages. It was effective in week 1 of treatment, maintained effects over several months, was well tolerated and safe, and showed improvement in sleep, behavior, and parenting stress.
Cortesi et al. (35)	134 (Melatonin = 34, CBT = 33, combined = 35, placebo = 32)	4–10	Melatonin	Randomized placebo-controlled trial	Melatonin treatment was mainly effective in reducing insomnia symptoms.
Wright et al. (36)	17 (Group A: Melatonin first, then placebo = 7; Group B: placebo first, then Melatonin = 10)	4–16	Melatonin	Randomized controlled crossover trial	Melatonin significantly improved sleep latency (by an average of 47 min) and total sleep (by an average of 52 min) compared to placebo, but not number of night awakenings.
Wirojanan et al. (37)	5 (Total sample = 12, including patients with FXS alone)	2–15	Melatonin, 2 weeks (placebo or Melatonin) and another 2 weeks (Melatonin or placebo) 3 mg/day	Randomized, double blind, placebo-controlled, crossover study	Mean night sleep duration was longer on melatonin than placebo by 21 min, mean sleep-onset latency was shorter by 28 min and mean sleep-onset time was earlier by 42 min. The results of this study support the efficacy and tolerability of Melatonin treatment for sleep problems in children with ASD and FXS.
Andersen et al. (38)	107	2–18	Melatonin	Retrospective and not placebo-controlled study	Melatonin appears to be a safe and well-tolerated treatment for insomnia in children with autism spectrum disorders.
Wasdell et al. (39)	16 (total sample = 50, including NDD); patients with neurological comorbidities were included	2–18	Melatonin	A randomized double-blind, placebo-controlled crossover trial followed by an open-label study	47 children with neurodevelopmental disabilities, who had treatment resistant chronic delayed sleep phase syndrome and impaired sleep maintenance, showed improvement in Melatonin therapy.
Garstang and Wallis (40)	7 (All patients took Melatonin for 4 weeks and placebo for other 4 weeks, sequence was randomly assigned)	4–16	Melatonin 9 weeks, with 4 weeks of trial (Melatonin or placebo), 1-week washout and other 4 weeks of trial (placebo or Melatonin) 5 mg/day	Randomized, placebo-controlled double-blind crossover trial	Melatonin significantly reduced sleep latency, number of nocturnal awakenings and increased the total sleep time compared to placebo, as assessed through sleep charts, daily completed by the parents. Sleep latency was 2.6 h baseline, 1.91 h with placebo and 1.06 h with melatonin. Wakings per night were 0.35 baseline, 0.26 with placebo and 0.08 with melatonin. Total sleep duration was 8.05 h baseline, 8.75 h with placebo and 9.84 h with melatonin.
Giannotti et al. (41)	20	2.6–9.6	Controlled-release Melatonin, 6 months (Follow-up: 2 years)	Open label, uncontrolled study	Sleep Diary and CSHQ showed problematic sleep in patients with ASD, with improvement after treatment with Melatonin in all subjects.
Galán et al. (42)	7 (Total sample = 22, including also 9 cerebral palsy and 6 ADHD)	6–14	Tryptophan-enriched cereals (200 mg of Tryptophan per 100 g), 5 weeks, (alternation of Tryptophan-enriched cereals and control cereals at dinner and breakfast)	Double-blind, placebo-controlled trial (placebo = control cereals with 75 mg of tryptophan per 100 g)	Higher sleep efficiency, lower sleep latency and total activity levels, measured with wrist actimeter, were registered when children with ASD ingested Tryptophan-enriched cereals at dinner, compared to control- and breakfast-weeks.

*6-GSI, 6-item Gastrointestinal Severity Index; ABC, Aberrant Behavior Checklist; ADHD, Attention-Deficit-Hyperactivity Disorder; ASD, Autism Spectrum Disorder; ATEC, Autism Treatment Evaluation Checklist; CARS2-ST, Childhood Autism Rating Scale, Second Edition—Standard Version; CBT, Cognitive Behavioral Teraphy; CSDI, Composite Sleep Disturbance Index; CSHQ, Children's Sleep Habits Questionnaire; FXS, Fragile X Syndrome; NDD, Neurodevelopmental Disorders; PedPRM, Pediatric Prolonged-Release Melatonin; PSQI, Pittsburgh Sleep Quality Index; SDQ, Strengths and Difficulties questionnaire; SMS, Smith-Magenis Syndrome; SND, Sleep and Nap Diary; SOL, Sleep-onset latency; TST, Total Sleep Time; VABS, Vineland Adaptive Behavior Scales; WHO-5, World Health Organization Well-Being Index.*

*,†Studies sharing same study design and participants.

## Results

Of the 21 articles included in final synthesis, 15 involved treatment with Melatonin, 1 study evaluated Tryptophan (Melatonin precursor) and was grouped to the studies concerning Melatonin, while the remaining five studies focused on supplementation with other antioxidant agent, namely Coenzyme Q10 (two studies), L-Carnosine (two studies), Luteolin and Quercetin (one study). Demographic characteristics, design and main results of the above-mentioned 21 studies are summarized in Table 1.

### Melatonin

As shown in Table 1B, sample size, treatment dose and trial design were very heterogeneous in the selected studies (28–41). Treatment with immediate-release Melatonin with doses varying from 2 to 10 mg/day was found to be well-tolerated and effective in shortening sleep-onset latency (SOL), reducing number of awakenings per night and bedtime resistance and in increasing total sleep time, as measured by sleep diaries (filled in by parents) (28, 32) or by actigraphy (33, 34). In those articles, a satisfactory improvement in sleep-related disturbances was obtained with low or medium (~1 to 6 mg/day) Melatonin doses in the majority of patients with ASD.

Both short- and long-term pediatric prolonged-release Melatonin (PedPRM) safety and effectiveness on sleep disturbances (as subjectively measured) with doses varying from 2 to 10 mg/day, were confirmed using randomized-placebo controlled trials (30, 31). Moreover, improvements in externalizing disruptive behaviors and parenting stress have been demonstrated with the same Melatonin formulation (29).

### Tryptophan

Tryptophan is an essential amino acid and a precursor for Melatonin. An actimetry study found higher sleep efficiency and reduced sleep latency and total activity levels in children with ASD when they were administrated with Tryptophan-enriched cereals at dinner, compared to period in which the participants received “control cereals” or Tryptophan-enriched cereals at breakfast (42).

### L-Carnosine

L-Carnosine is a natural dipeptide (b-alanine and L-histidine) concentrated in brain and muscles, acting as an antitoxic and neuroprotective agent, whose circulating levels were recently found to be lower in patients with ASD than in control subjects (6).

Recently, two different research groups specifically investigated the impact of L-Carnosine supplementation on sleep disturbances in children with ASD, as reported by parents, using subjective measures (23, 24). In one, double-blind, randomized trial a statistically significant reduction in Children's Sleep Habits Questionnaire (CSHQ) scores and specifically in “sleep duration,” “parasomnias” and “total sleep disorders” was found at the end of intervention in children and adolescents with ASD receiving L-Carnosine compared to the placebo group (24). A subsequent, non-blinded trial on pre-school patients was not able to replicate these results using the BEARS sleep screening tool (23).

### Coenzyme Q10 (Ubiquinone and Ubiquinol)

Coenzyme Q10 is a lipid-soluble benzoquinone involved in oxidative phosphorylation as a cofactor for enzyme complexes in the mitochondrial membrane. It also has a recognized role as a free radical scavenger (44). Two studies have investigated the effect on sleep disturbances in ASD patients after supplementation with, respectively, Ubiquinone (Coenzyme Q10 oxidized form) and Ubiquinol (reduced form) (25, 26). In particular, Mousavinejad and coauthors designed a placebo-controlled trial with a relatively large sample (78 patients) and found significantly higher rate of improvement in sleep disorder, as subjectively reported by parents, in patients treated with high-dose (60 mg/day) Ubiquinone compared to placebo and to low-dose treatment groups (25). Improvements in parent-reported sleep disturbances after Ubiquinol supplementation were also reported in the 34% of patients in a previous study (26).

### Luteolin and Quercetin

One of the selected studies investigated the effectiveness and tolerability of combined dietary supplementation with two flavonoids, Luteolin and Quercetin, finding a significant improvement in adaptive functioning, measured with Vineland Adaptive Behavior Scales (VABS) scores, and in overall behavior, with a reduction in Aberrant Behavior Checklist (ABC) subscale scores (27). Effect on sleep problems was not addressed as an outcome, while “sleep difficulties” were reported as adverse effect of the treatment in 3/40 patients (27).

## Discussion

Increased levels of oxidative stress markers in individuals with disturbed sleep and also in patient with ASD are known by the literature (5, 6), confirming their possible role in the pathogenesis of sleep disturbances as well as ASD (10). Despite the growing amount of literature and research interest on effect of antioxidant agents on ASD, most of the data focuses on Melatonin supplementation. Moreover, the effect of antioxidants on sleep problems remains poorly investigated as a primary endpoint, being seldomly cited among the various behavioral aspects evaluated for improvement.

Literature data highlight evidence for an effect of Melatonin (and Tryptophan) on sleep in children with ASD as reported in Table 1B. The relatively low number of articles concerning treatment with Melatonin covered by this article is mainly due to the choice of keywords and search strategy, which allowed the inclusion of only those papers reporting “antioxidant” or “oxidative stress” in full text, in any field.

The supplementation with both immediate- and prolonged-release Melatonin was found to be effective for treatment of insomnia in children and adolescent with ASD, determining improvements in sleep latency, total sleep time and reduced the number of nocturnal awakenings in subject with ASD, as measured prevalently by subjective (scales/questionnaire/diaries) tools (28–30, 32) and confirmed by some actigraphic findings (33, 34). Melatonin is also considered a safe and well-tolerated medication in the dose range of 2–10 mg/day in children and adolescents.

It should be highlighted that Melatonin is reported to play a key role in multiple pathways (circadian rhythm regulation, anti-inflammatory and antioxidant properties); the effectiveness of Melatonin on sleep improvement in children with ASD may therefore be the result of different and maybe synergic mechanisms.

With regard to the other antioxidant agents included in our review, the study on Tryptophan dietary implementation showed promising results despite the extremely small sample size (42) and should therefore be addressed for future investigations.

Evidence that supplementation with L-Carnosine could determine an improvement in sleep troubles is limited to one study: Mehrazad-Saber et al. documented a significant reduction in “Total sleep disorders,” “Sleep duration” and “Parasomnias” scores assessed by CSHQ in a group of 21 children and adolescents treated with L-Carnosine 500 mg/day compared to control subject. On the other hand, Ann Abraham et al. failed to find improvement in sleep disturbances as assessed by BEARS sleep screening tool. Possible explanations for these contrasting results might be found in the different dose of L-Carnosine (higher in the first study), in the possible interference/synergy of concomitant chronic pharmacological treatments (excluded in the second study) but also it might be due to methodological differences, in terms of scales used to investigate the presence/severity of sleep disorders in the two samples.

Beneficial effects of Coenzyme Q10 on sleep in patients with ASD was primarily demonstrated by Gvozdjáková et al. in a group of 24 children, Ubiquinol supportive therapy was effective in improving sleep troubles in 34% of patients (26). Anyway, the small sample size and the study design (absence of a placebo group) constitute the main limitations of the study. More recently, Mousavinejad et al. documented the effectiveness of high doses of Coenzyme Q10 in improving subjectively reported sleep disorders in children with ASD when compared to placebo and low-dose treatment (25).

Luteolin and Quercetin combined effect was evaluated in one study only and failed in demonstrating a beneficial effect on sleep (27).

Data regarding the effects of antioxidant agents, other than Melatonin, on sleep are sparse and/or not well-documented. Main limitations of the included studies were: the usually small sample size (compared to ASD prevalence in general population), the heterogeneity of the used scales and the questionnaires (frequently investigating patients' global functioning and only seldomly focusing on sleep habits) and the lack of objective measurement of sleep.

It could be speculated that antioxidant agents that were documented to have beneficial effects on behavioral aspects could also act on sleep disturbances, characterized by bedtime resistance or difficulty in initiating sleep. In this context it could be interesting to evaluate the effects of other molecules, such as Sulforaphane, whose effectiveness on improving behavioral symptoms has already been demonstrated (21), on sleep outcome.

It is well-known that a correct diagnosis and treatment of the pathophysiological comorbidities of ASD can lead to a better outcome (45). It is therefore necessary to primarily identify some tools that could be used for the screening of sleep problems and that allow for a better characterization of the sleep disturbances. It is also fundamental to identify the candidates to further instrumental investigations, such as polysomnography and referral to a sleep medicine center.

In addition to scales and questionnaires already used in the management of subjects with ASD it could be useful to develop more specific tools for sleep disturbances evaluation in these subjects. Currently specific scales or validated diagnostic algorithms are not available and sleep troubles are evaluated with different methods including both subjective and objective methods.

In conclusion, despite the high prevalence of sleep troubles in children and adolescents with ASD there is a paucity of data looking at the efficacy of antioxidant agents in this group of subjects.

More research is needed to better define and the role of antioxidants agents as adjunctive therapy in the management of children and adolescents with ASD affected by sleep disorders.

## Author Contributions

EZ and MPC contributed to conception and design of the study. AL and EZ wrote the first draft of the manuscript. AL, EZ, and KT wrote sections of the manuscript. SS participated in the analysis of the data. All authors contributed to the article and approved the submitted version.

## Conflict of Interest

The authors declare that the research was conducted in the absence of any commercial or financial relationships that could be construed as a potential conflict of interest.
